# Help seeking and suicidality among people with epilepsy in a rural low income country setting: cross-sectional survey

**DOI:** 10.1186/s13033-017-0151-5

**Published:** 2017-07-14

**Authors:** Ruth Tsigebrhan, Charlotte Hanlon, Girmay Medhin, Abebaw Fekadu

**Affiliations:** 1 Department of Psychiatry, School of Medicine, College of Health Sciences, Addis Ababa University, Addis Ababa, Ethiopia; 2 Centre for Global Mental Health, Health Services and Population Research Department, Institute of Psychiatry, Psychology and Neuroscience, King’s College London, London, UK; 3 Aklilu Lemma Institute of Pathobiology, Addis Ababa University, Addis Ababa, Ethiopia; 4 Centre for Affective Disorders, Department of Psychological Medicine, Institute of Psychiatry, Psychology and Neuroscience, King’s College London, London, UK

**Keywords:** Suicidality, Help seeking, Untreated epilepsy, Depression, Low income country

## Abstract

**Backgrounds:**

Epilepsy is a serious neurological disorder associated with a high level of psychiatric comorbidity. Suicidality is a recognised complication of epilepsy. As part of developing an integrated service for people with epilepsy (PWE) and priority psychiatric disorders within primary care, a cross-sectional study was conducted in a rural district in Ethiopia to investigate patterns of help-seeking, suicidality and the association with duration of untreated epilepsy (DUE) among PWE.

**Methods:**

Cases were identified through community key informants and diagnosis was confirmed by trained primary care clinicians. Severity of epilepsy, depression and suicidality were assessed using standardised methods. Multivariable regression analysis was used to test the hypothesis that suicidality was associated with DUE.

**Results:**

The majority of PWE sought help from both religious and biomedical healing centres. The lifetime treatment gap for biomedical care was 26.9%, with a 12 month treatment gap of 56.7%. Close to one-third (29.9%) of participants reported using traditional and cultural healing practices. Nearly one-third (30.2%) of participants reported suicidality (suicidal ideation, plan or attempt) in the previous 1 year. The median (IQR) DUE was 24 months (4–72). There was no association between DUE and suicidality. In the multivariable model, being married [odds ratio (OR) 2.81, 95% CI 1.22, 6.46], increased depressive symptoms (OR 1.17, 95% CI 1.10, 1.26) and perceived poorer wealth relative to others (OR 2.67, 95% CI 1.07, 6.68) were associated independently with suicidality.

**Conclusion:**

In this study, PWE sought help from both biomedical and religious healing centres. Suicidality and depression have a high prevalence in PWE in this setting. Integrated mental and neurological health care within primary care is needed for improved holistic management of epilepsy.

## Background

Epilepsy can be a chronic and disabling disorder which has an impact on social, interpersonal and occupational functioning [1]. Globally, an estimated 65 million people are affected by epilepsy [2]. In low- and middle-income countries (LMICs), the median estimate for lifetime prevalence of epilepsy is 15.4 per 1000 people in rural areas and 10.3 per 1000 in urban settings [3]. Epilepsy is estimated to have resulted in 116 deaths per 100,000 people worldwide in 2013 [4]. In Ethiopia, the prevalence of epilepsy was 520/100,000 people in a large-scale, rural, community-based survey [5].

Studies from high- and middle-income countries indicate an increased risk of psychiatric comorbidity among people with epilepsy compared to the general population [2, 6]. Hospital based studies conducted in Ethiopia have shown that the prevalence of depression is high among people with epilepsy [7–9]. Comorbid psychiatric disorders, such as depression, have a substantial negative impact on quality of life [10–12] and are important risk factors for suicidal behaviours [13]. In large case control studies from Sweden and Denmark, the odds of suicide were increased up to threefold in people with epilepsy compared to the general population [14, 15].

In LMICs, the proportion of people receiving basic treatment for epilepsy is limited [3, 16]. In a general population and hospital-based survey conducted in 12 African countries, a lifetime treatment gap of 30.6% was identified, with 11.7% of the respondents using traditional healing exclusively [17]. In a rural African community, the mean duration of epilepsy before receiving evidence-based biomedical treatment was six and half years [18]. The simultaneous use of biomedical and traditional treatments for epilepsy has been found to be commonplace [19, 20]. A reliance upon traditional healing approaches has also been observed in Ethiopia [21]. In some situations traditional and religious treatment modalities may be the only choice available, with an absence of accessible biomedical services for people with epilepsy [22].

There have been few studies from low-income country contexts to investigate the help-seeking behaviour of people with epilepsy (PWE) in relation to suicidality. Undetected and untreated epilepsy is expected to be associated with higher psychiatric morbidity and elevated suicidality. Evidence is needed to inform current plans to scale up the integrated management of people with mental and neurological disorders in LMICs and to reduce the delay in people accessing treatment.

The aim of this study was to estimate the time delay between first seizure and initial receipt of evidence-based treatment (duration of untreated epilepsy) and to explore the association with suicidality (suicidal idea, plan or attempt). We hypothesized that suicidality in people with epilepsy would be associated with longer duration of untreated epilepsy.

## Methods

### Design

Cross-sectional survey of community-ascertained cases of PWE attending a newly implemented primary care-based epilepsy service.

### Setting

The study was carried out as part of the programme for improving mental health care (PRIME), a research programme consortium across five LMICs (Ethiopia, Nepal, India, South Africa and Uganda) [23]. PRIME Ethiopia is currently implementing a programme of care for people with epilepsy, psychosis, bipolar disorder, depression and alcohol use disorders integrated into primary health care in the Sodo district of Ethiopia [24]. Sodo district is located in the Gurage Zone, 100 km south of the capital city, Addis Ababa, with a population of 161,952 people at the time of the 2013 census [25]. The district is predominantly rural and is classified administratively into 58 sub-districts (*kebeles*), the lowest government administrative structure. Amharic is the official language of the Sodo district. There are a total of eight primary care health centres and 54 health posts (community based health facilities) available in the district. However, at the time of the study, there was no formal mental health or neurological care provided within the district. The nearest service was the nurse-led psychiatric unit in Butajira hospital and a neurological clinic (*“Grarbet”*) which is 30 km away from the centre of Sodo district [25, 26].

### Study population and sampling

Data were collected from adults who had been identified as having possible epilepsy by trained community key informants, referred to primary care and then confirmed to have convulsive epilepsy by a primary health care clinician. This key informant method was previously found to be effective for psychosis case ascertainment in the neighboring district [27]. Primary care-based treatment was offered according to the evidence-based treatment guidelines of the World Health Organisation’s mental health Gap Action Programme Intervention Guide (WHO mhGAP-IG) [28]. The inclusion criteria were (1) age ≥18 years, (2) mhGAP definition of a convulsive seizure: *“recurrent (at least twice) unprovoked seizure occurring days or weeks apart”.* and (3) resident in Sodo district. The assessment recommended by mhGAP-IG includes neurological examination but does not require an electroencephalogram (EEG). The first thirty cases (10%) were assessed by a neurologist for confirmation of the diagnosis. There was 100% concordance between the epilepsy diagnosis made by the primary health care clinicians and that made by the neurologist.

The target sample size of 300 people was determined for the PRIME intervention study [29]. The sample size was adequate to detect a 6 month difference in DUE between those with and without suicidality with the following assumptions: DUE among those with suicidality is 70 months and those without is 64 months, standard deviation of 15, 90% power, 95% confidence and 10% contingency.

### Measurements

#### Primary outcome: suicidality

Suicidality was defined as the presence of one of the following: suicidal ideation or suicidal plan or suicide attempt in the past 12 months. Suicidality was measured using the suicide module of the composite international diagnostic interview (CIDI) [30]. The acceptability, reliability and feasibility of the Amharic version of CIDI was evaluated in the Ethiopian setting previously [31].

#### Primary exposure: duration of untreated epilepsy (DUE)

DUE was defined as the time between the first seizure and the first receipt of evidence-based treatment (in months) measured by participant self-report. Evidence-based treatment was defined according to WHO’s mhGAP-IG [28].

Potential confounding variables

- Socio-demographic characteristics: age, sex, education, marital status, relative wealth, area of residence.
- Types of seizure disorder [focal seizures (FS) and generalized tonic–clonic (GTC)] as diagnosed by the primary health care clinician.
- Severity of epilepsy: measured using the national hospital severity scale (NHS3) which is an eight item questionnaire to evaluate the nature and consequences of seizures during the preceding 6 months [32]. The higher the score the greater the severity of epilepsy. Collateral information was obtained from attendants where possible.
- Help seeking patterns were measured by asking whether the person had sought treatment from any healing places for their seizure and the type of treatment they had used. The types of traditional and religious treatments used in the setting are diverse [33]. The help sought was categorized as traditional, religious, biomedical or no treatment at all.
- Accessibility of care, measured in terms of the average time to reach the nearest health centre (in minutes).
- Alcohol use disorders was measured using the Alcohol Use Disorder Identification Test (AUDIT) [34]. The AUDIT has been adapted for Ethiopia [35], with support for the recommended cut off scores for hazardous use (≥8), harmful use (≥16 points) and dependent drinking (≥20 points).

Depression was hypothesized to be on the causal pathway (a potential mediator) between DUE and suicidality. Depressive symptoms were measured using the total score on the patient health questionnaire (PHQ-9).

The PHQ-9 is based on the DSM-IV diagnostic criteria for major depressive disorder and has been used to screen adults (not including PWE) for depression with an optimal cut off score 10 in clinical and community settings [36]. Categories of PHQ score have been used to define levels of depression [37]: no depression for a PHQ-9 score of 0, mild depression for PHQ score from 1 to 9, moderate depression for PHQ score from 10 to 14 and severe depression for a score of 15 or above. The Amharic version of the PHQ-9 has been validated in primary care settings in rural Ethiopia, with a score of five or more optimal for indicating risk of depression among adults without epilepsy [38]. As the PHQ-9 has not been validated in PWE in Ethiopia we used both cut-offs.

Family size was considered to be an indicator of social support and conceptualised as a potential effect modifier of the association between DUE and suicidality.

### Data collection

All lay interviewer-administered measures were translated into the local language (Amharic). The lay data collectors were trained on the administration of the instruments for 1 week by psychiatric nurses and PRIME research assistants (Master’s level). Training included role play and observed interviews with volunteers. The confirmatory clinical evaluation of epilepsy and the epilepsy severity scale, both of which are observer-rated scales, were completed in English by primary health care clinicians. Close supervision was provided by the project employees and psychiatric nurses.

### Data analysis

Data were double entered using Epi-data version 3.1 and analysed using STATA version 12. Simple descriptive analyses were used to summarise the socio-demographic and clinical characteristics of the study participants. Help seeking patterns and type of treatments used by the study participants were calculated as percentages and their association with suicidality was assessed using Pearson Chi square or Fisher’s exact test as appropriate. The median DUE was calculated by including the people who had sought previous evidence-based medical treatment combined with those people who accessed their first biomedical treatment through PRIME. Hypothesis driven analysis of the association between DUE and suicidality was carried out using multiple logistic regression modelling, adjusting for confounders identified a priori. Multivariable logistic regression was conducted, including only the variables which were associated with suicidality (at the level of p < 0.2) in the univariate analysis. A sensitivity analysis was also conducted with the suicide item removed from the PHQ-9 total score. Exploration of a mediational role of depressive symptoms was planned by adding the PHQ score into the multivariable model. Exploration of effect modification by family size was also done.

## Result

A total of 298 people with epilepsy were assessed. All of the participants with probable epilepsy who had been detected and referred from the community attended the designated health centre. As shown in Table 1, the majority were male (58.7%) and had no formal education (60.3%). Around one-third were less than 25 years of age (34.0%) with a mean age of 33.3 years (SD = 13.7). Almost all participants (90.6%) were living in rural areas but residing within two hours travel of their nearest health centre. Nearly two-thirds of participants perceived their socioeconomic status to be low or very low relative to others (65.0%). Family size ranged from one up to twelve members, with an average of three children per household (Table 1).

**Table 1:** Socio-demographic characteristics

Characteristics (total N = 298)	Number (%)
Age (years)	
<25	101 (34.0)
25–34	71 (23.9)
35–44	62 (20.9)
≥45	63 (21.2)
Mean age (standard deviation; SD)	33.3 (13.7)
Gender	
Male	175 (58.7)
Female	123 (41.3)
Education	
No formal education	178 (60.3)
Formal education	118 (39.7)
Employment	
Employed	37 (12.5)
Unemployed	55 (18.6)
Farmer	102 (34.6)
House wife	57 (19.3)
Others*	45 (15.2)
Area of residence	
Rural	270 (90.6)
Urban	28 (9.4)
Perceived relative wealth	
Very low	50 (16.8)
Low	143 (48.2)
Average and above	104 (35.0)
Marital status	
Single	138 (46.3)
Married	129 (43.3)
Formerly married	31 (10.4)
Religion	
Orthodox christian	275 (92.2)
Protestant	13 (4.4)
Muslim	7 (2.4)
Others	3 (1.0)
Ethnicity	
Gurage	278 (93.3)
Oromo	17 (5.7)
Amhara	2 (0.7)
Others**	1 (0.3)
Mean family size (SD)	5.5 (2.1)
Mean number of children (SD)	3.2 (2.4)
Duration of travel to reach the nearest health center (min)	
0–30	*120 (40.8)*
31–120	*141 (48.0)*
>120	33 (11.2)

* Includes students, pensioners and volunteer workers

** Others include Mareko, Meskana, Kebena

### Epilepsy diagnosis, severity and duration of untreated illness

Almost all (93.0%) of the participants were diagnosed with generalized tonic–clonic seizures (GTC) (Table 2). The mean epilepsy severity scores for GTC and FS were not meaningfully different. The age of onset of seizures ranged from less than 1 year up to a maximum of 70 years, with a median age of onset of 17 years (IQR 10–29). The median total duration of illness was 10 years (IQR 4–18.5). The lifetime treatment gap was 26.9% and the 12 month treatment gap was 56.7%. The median DUE was 24 months (IQR 4–72) (Table 2).

**Table 2:** Clinical characteristics of people with epilepsy attending primary care (N = 298)

Clinical variable	Response categories	Number (%)
Types of epilepsy	Focal seizure	21 (7.0)
	Generalized tonic–clonic	277 (93.0)
Median age of onset (IQR)		17.0 (10–29)
Median duration of illness (IQR)		10 (4–18.5)
Epilepsy severity score [mean NHS3 score (SD)]	Focal seizure	10.0 (4.6)
	Generalised tonic–clonic seizure	10.6 (3.5)
Suicidality	Suicidal ideation	90 (30.2)
	Suicidal plan	47 (15.8)
	Suicidal attempt	28 (9.4)
Depressive symptoms*	No depression	13 (4.4)
	Mild depression	181 (60.7)
	Moderate depression	80 (26.8)
	Severe depression	24 (8.1)
Alcohol use disorder	No alcohol use	217 (72.8)
	Hazardous	48 (16.1)
	Harmful	8 (2.7)
	Dependent	25 (8.4)
Median DUE (IQR)		24 (4–72)

*IQR* inter quartile range, *AUDIT* Alcohol Use Disorder Identification Test, *NHS3* national hospital severity scale, *SD* standard deviation *PHQ*-*9* patient health questionnaire, *DUE* duration of untreated epilepsy

* Categories defined by PHQ scores as described in the methods

### Suicidality and psychosocial characteristics

One-third of the study participants (30.2%) reported suicidal ideation during the preceding 12 months, 15.8% had a suicide plan and 9.4% had attempted suicide.

The prevalence of possible depression was 70.8% using the cut-off of 5 or more, and 34.9% using the cut-off of 10 or more. Over a quarter of participants had probable alcohol use disorder (27.2%). The median DUE was 24 months for both participants with suicidality (IQR 8–84) and non-suicidality (IQR 3.5–72).

### Help-seeking for epilepsy

All participants who reported seeking help from non-biomedical or biomedical sources attended one or more religious settings (churches, monasteries, mosques and holy water places). Help seeking patterns are described in supplementary table one. Nearly one-third (29.9%) of participants reported using traditional and cultural healing practices. Help-seeking from churches was higher in people expressing suicidality compared to those without (51.1% vs. 31.3%; X^2^ 10.6, df = 1, p = 0.001) and similarly for those attending monasteries (22.2% vs. 11.5%; X^2^ 5.70, df = 1, p = 0.02). There was no significant difference in the mean epilepsy severity score of those participants with GTC and attending churches compared to those who did not (p = 0.80) or between those attending monasteries compared to non-attenders (p = 0.98).

### Duration of untreated epilepsy and suicidality

There was no significant association between DUE and suicidality in univariate analysis [odds ratio (OR) 0.85, 95% confidence interval (CI) 0.50, 1.44] or after adjusting for hypothesised confounders (OR 3.38, 95% CI 0.59, 19.34). Even if there was a change of odds ratio of DUE in the multiple logistic regression after using the interaction term, this was not significant. In view of this finding, no test for mediation by depression was conducted.

### Multivariable analysis of factors associated with suicidality

The adjusted odds of suicidality among PWE were statistically significantly higher among people who were married (OR 2.81, 95% CI 1.22, 6.46), had higher levels of depressive symptoms (OR 1.17 for each 1 point increase in PHQ-9 score, 95% CI 1.10, 1.26) and those with perceived very low relative wealth (OR 2.67, 95% CI 1.07, 6.68) in the final multivariable model (Table 3). In the sensitivity analysis, the association of suicidality with depression was unchanged after the suicide item was removed from the PHQ-9 questionnaire (adjusted OR 1.16 for each 1 point increase in PHQ-9 score, 95% CI 1.09, 1.23). Even though family size was hypothesized to be an effect modifier, the interaction between DUE and family size was non-significant (p = 0.10) (Table 3).

**Table 3:** Factors associated with 12 months suicidality (dependent variable) in people with epilepsy (n = 298)

Characteristic (independent variables)	Suicidality number (%)	Univariate analysis		Multivariable analysis	
		Crude odds ratio (OR) for suicidality	95% Confidence interval (CI)	Adjusted OR for suicidality	95% CI
Age (in years)		1.01	0.99, 1.02	0.99	0.95, 1.02
Gender					
Male	44 (14.7)	1.00		1.00	
Female	46 (15.4)	*1.78*	*1.08*, *2.93*	1.61	0.87, 2.98
Marital status					
Single	33 (11.1)	1.00		1	
Married	46 (15.4)	*1.76*	*1.04*, *3.00*	*2.75*	*1.19*, *6.35*
Formerly married	11 (3.6)	1.75	0.76, 4.02	1.56	0.46, 5.26
Relative wealth					
Average and above	21 (7)	1.00		1.00	
Poor	49 (16.4)	*2.04*	*1.14*, *3.72*	1.64	0.80, 3.33
Very poor	19 (6.4)	*2.42*	*1.15*, *5.10*	*2.67*	*1.07*, *6.68*
Education					
No formal	56 (18.8)	1.00			
Formal education	34 (11.4)	0.89	0.53, 1.48		
Area of residence					
Urban	9 (3)	1.00			
Rural	81 (27.2)	0.90	0.39, 2.08		
Family size		0.94	0.83, 1.06	1.03	0.84, 1.27
Duration of travel to reach the nearby health center (minutes)					
0–30	33 (11.1)	1.00			
31–120	43 (14.4)	1.16	0.68, 1.98	0.89	0.47, 1.72
>120	13 (4.4)	1.71	0.77, 3.83	1.16	0.42, 3.15
DUE (in months)					
0–23.99	38 (12.8)	1.00		1.00	
24–636	40 (13.4)	0.85	0.50, 1.44	3.24	0.56, 18.86
Biomedical treatment					
Yes	68 (22.8)	1.00			
No	22 (7.4)	1.20	0.68, 2.11		
Epilepsy severity (total NHS3 score)					
FS		1.09	0.99, 1.19		
GTC		1.03	0.97, 1.12		
Depressive symptoms (total PHQ-9 score)		*1.18*	*1.11*, *1.25*	*1.17*	*1.10*, *1.26*
Use of alcohol					
No use	64 (21.5)	1.00			
Hazardous	17 (5.7)	1.31	0.68, 2.54		
Harmful	2 (0.7)	0.80	0.16, 4.05		
Dependent	7 (2.3)	0.93	0.37, 2.33		

*AUDIT* Alcohol Use Disorder Identification Test, *NHS3* national hospital severity scale, *PHQ*-*9* patient health questionnaire, *DUE* duration of untreated epilepsy, *GTC* generalized tonic seizure, *FS* focal seizure, *HC* health centre

## Discussion

In this cross-sectional study of community-ascertained PWE about to receive primary care-based treatment in rural Ethiopia, suicidality was present in nearly one-third of PWE, and was associated independently with increased depressive symptoms, being married and having low socio-economic status. The 12 month treatment gap for epilepsy was high (56.7%) and the median duration of untreated epilepsy was long (24 months). The findings of this study did not support the hypothesis that longer DUE was associated with increased suicidality.

The high prevalence of suicidality in PWE in our study is in keeping with findings from studies conducted in high income countries [14, 15, 39]. The prevalence of suicidality in PWE in this study was also higher than the prevalence in the general population in Sodo district (20.5%) [40]. A previous suicide attempt is one of the major risk factors for future completed suicide and, as such, is an important target for suicide prevention efforts [39]. Nearly one in ten PWE in our study had attempted suicide in the preceding 12 months. This result underlines the importance of assessment for suicide risk in PWE and interventions to reduce risk. The high levels of stigmatising attitudes towards both epilepsy and mental health problems may complicate early recognition of suicide risk and interventions to prevent suicide in this rural community [26, 41, 42]. In a previous study conducted in a rural district neighbouring the setting for the current study, only 21.5% of key informants considered mental illness to be possible cause of suicide [43]. Once a person has attempted suicide, this brings further stigma due to prohibitions against suicide an all of the major faiths in Ethiopia [43].

In agreement with research from other non-African countries we found an association between both depression [15, 39, 44] and low socioeconomic status [45] with suicidality. Furthermore, depression was found to be associated with suicidality independently of seizure severity [13]. The prevalence of depression among PWE in this resource limited setting using the primary care validated cut-off (70.8%) was also higher than the general population depression prevalence (28.8%) using the same validated cut-off (5 or more) [38]. This prevalence of depression was also higher than the prevalence that is reported in the institution based studies from Ethiopia [7–9]. Various explanations have been proposed to explain the elevated levels of depression in epilepsy; (1) underlying structural brain disease, (2) damage related to uncontrolled seizures, and (3) psychosocial impact of the stigma and disability associated with epilepsy [6]. Validated cut-off scores to indicate possible depression in primary care populations in general (mostly with acute infectious conditions) may not be valid in persons with chronic disorders. However, even with a more conservative cut-off score (10 or more), more than one-third of PWE had high levels of depressive symptoms. These high levels of depression could also be related to the high prevalence of alcohol use in this population. In previous studies, co-morbid depression in PWE has been demonstrated to have a substantial negative impact on quality of life [10, 13] and contributes to greater disability than attributed to seizures [12] and to increased risk of suicide [15, 45].

The association of suicidality with being married is inconsistent with other studies [45] but this unusual association has been also observed in the general population of Sodo district [40]. The possible explanation for this association has been proposed as increased conflict in married couples [40]. In general, people with epilepsy in our study were less likely to be married (only 50%) compared to the general population (74.8%) [40]. It is likely that the low prevalence of marriage is related to stigma, poor seizure control and misunderstanding of the cause of epilepsy. It is also possible that in people with epilepsy who do get married (or who develop epilepsy after marriage) there may be more direct exposure to stigmatising attitudes and discrimination from the family of the spouse, which could explain the higher levels of suicidality and the rates of discrimination and disability related to epilepsy could be much worse for those who are married than the singles. This hypothesis needs further exploration of the perception of married PWE on stigma and discrimination in this rural setting.

The overall prevalence of alcohol use disorders (27.2%) was high in this community sample of people with epilepsy and it was even higher than the general Sodo district population prevalence (22.6%) [40]. Alcohol use disorders have a causal role in the development of epilepsy which may explain the elevated levels of alcohol use disorders; however, the stigma and elevated mental health problems associated with epilepsy may also lead to increased risk of substance use. A prospective study is needed to investigate the direction of association. The lack of association between AUDs and suicidality was unexpected.

Although the median DUE was 2 years, it was lower (by 2 years) than that found in a study conducted in northern Ethiopia 16 years ago [22]. The average DUE in this study was also lower than the findings from another African low income country (6.5 years) [18]. This apparent decline in DUE over time could be due to the availability of a neurological clinic (‘*Grarbet*’) in the neighbouring town, although over half of PWE still had not accessed evidence-based treatment in the preceding year [26]. This suggests that many participants who had initiated evidence-based treatment are likely to have had subsequent prolonged periods of non-treatment, leading to underestimation of the impacts of delayed biomedical treatment on suicidality. The wide confidence interval and the lack of association between DUE and suicidality would have been also minimized with bigger sample size. Furthermore, the measurement of self-reported DUE could have been affected by recall bias, which might have been expected to bias the finding to the null.

The treatment gap identified for epilepsy in this rural district of Ethiopia is comparable to the result of a multi-country African study [17], but lower than the findings in studies from Ethiopia [46] and other low income countries [16, 19, 47]. A number of possible causes of treatment gap have been found in the Sub-Saharan African countries including inconsistent availability and unaffordability of anti-epileptic medications, stigma, inadequate skilled health professionals, limited access to health institution, medication side effects and toxicity [16, 47].

A study from Ethiopia found a high percentage (12%) of default from epilepsy treatment because of the preference for traditional remedies [21]. Therefore, besides the need to expand local access to low cost anti-epileptic drugs, belief systems about the causes of epilepsy, comorbid psychiatric disorders and the acceptability of biomedical treatments may be important in understanding the factors playing in treatment adherence. This study has also demonstrated the presence of widespread use of religious treatment facilities among the study participants who are treated with or without the addition of evidence based medicine, in keeping with other studies [17, 20]. The attribution of causes of epilepsy to supernatural forces might have also influenced the preference for religious help seeking in participants [33]. Providing mental health education and integrating traditional healers of the rural communities of Ethiopia into the contemporary biomedical treatment could facilitate the referral system to medical centres and narrow the treatment gap.

Integration of mental health services at the primary health care level is likely to further enhance detection and management of co-morbid mental health problems in PWE. Our findings indicate that PWE may benefit from the co-ordinated scale-up of integrated services for mental health and neurological conditions, due to the high levels of co-morbid depression and suicidality. The World Health Organisation’s mhGAP trains primary care workers in evidence-based guidelines for suicide, depression and epilepsy. However, the reliance on psychotropic medication to treat depression in this setting may not be acceptable to PWE who are already taking long-term medications [24]. There is a need to adapt and expand the availability of psychosocial interventions for depression in PWE, combined with poverty reduction programs to address the social determinants of mental health problems in this setting.

The inclusion of community-ascertained cases, using rigorous methodology which has been used in this rural Ethiopian context previously, is a strength of this study [27]. The pragmatic design with very few exclusion criteria ensured representativeness of the sample and generalisability to other rural areas of Ethiopia, Standardised and validated measures were used which had robust psychometric properties. The primary hypothesis could only have been examined with a cross-sectional analysis due to the ethical imperative to provide treatment to people identified as having epilepsy. Even though the majority of measurements relied upon self-report questionnaires, information bias (recall bias) was minimized by also interviewing the attendants of the participants.

The main limitation of the study was the cross-sectional design. Therefore, it was not possible to investigate causality or the temporal relationship between untreated epilepsy and suicidality, or the temporal relationship between religious healing and suicidality. A prospective study and qualitative exploration of the individual’s perspectives on religious healing are needed. In addition to the clinical diagnosis, standardised approaches to diagnosing epilpsy could have increased the validity of the study. Although we included a wide range of potential confounders in the hypothesis testing multivariable analysis looking at duration of untreated epilepsy and suicidality, other important factors (e.g. presence of other psychiatric disorders such as anxiety and psychosis) may have been relevant. There is also potential limitation in this study for not analysing sub-categories of suicidality. The nature of the sampling procedure and our sample size may have limited our power to detect true associations. The finding from this paper may not be applicable to the urban parts of Ethiopia since the socio-demographic and health related factors are different from the rural area.

## Conclusions

In this study of PWE in a rural Ethiopian community, the prevalence of suicidality was high and associated strongly with depressive symptoms. Integrated management of epilepsy and associated mental health conditions at the primary care level, as recommended by the WHO, has the potential to improve the holistic treatment, reduce suicide risk and enhance the quality of life of PWE. However, reducing the epilepsy treatment gap requires attention to preferred help-seeking practices in this rural, non-Western society.

## Abbreviations

AUDIT: Alcohol Use Disorder Identification Test
CIDI: composite international diagnostic interview
DUE: duration of untreated epilepsy
FS: focal seizure
GTC: generalized tonic–clonic
ICD: international classification of diseases
IQR: inter quartile range
LMICs: low and middle income countries
NHS3: national hospital severity scale
PHQ-9: patient health questionnaire
PRIME: programme for improving mental health care
PWE: people with epilepsy
WHO mhGAP-IG: World Health Organisation’s mental health gap action programme intervention guide
